# Supplementary material for: Ni/Mn-Complex-Tethered Tetranuclear Polyoxovanadates: Crystal Structure and Inhibitory Activity on Human Hepatocellular Carcinoma (HepG-2)
Source: Molecules. 2023 Sep 28;28(19):6843. doi: 10.3390/molecules28196843 (PMC10574323; doi:10.3390/molecules28196843)
Supplement: Supplementary file 1 [file molecules-28-06843-s001.zip › molecules-2604251-supplementary.pdf]

Supplementary Materials

Experimental section

General characterization

The Fourier transform infrared (FT-IR) spectra were collected in the range of 4000-400 cm^{-1} on a Nicolet 170SXFT/IR spectrometer with KBr pellets dispersed samples. The powder X-ray diffraction (PXRD) pattern on the samples were recorded on a Rigaku D/max-2550 diffractometer with Cu-K_α radiation (λ = 0.154 nm) at room temperature. All crystallographic data were recorded at 298 K on a Bruker APEX-II CCD detector with graphite monochromatic Mo-K_α radiation (λ = 0.71073 Å). The Elemental analyses (C, H and N) were performed on a Perkin Elmer 240C elemental analyzer.

Crystallography

Crystal data for POVs **1-2** were collected on Rigaku RAXIS RAPID IP with Mo-K_α monochromatic radiation (λ = 0.71073 Å) at 298.15K. The structures were solved by the direct methods and refined by full-matrix least-squares refinements based on F^2 using the SHELXS-2014 and SHELXL-2014 programs [47]. All the non-hydrogen atoms were refined anisotropically. The positions of hydrogen atoms on carbon atoms were calculated theoretically. The crystallographic data have been deposited with the Cambridge Crystallographic Data Center (CCDC) as entries 2279426 (**1**), 2279427 (**2**). A summary of the crystal data, data collection and refinement parameters for **1-2** are given in Table S1. Selected bond lengths and bond

angles of POVs **1-2** are summarized in Table S2.

Table of contents

1. Table S1 Crystallographic data for POVs **1-2**

2. Table S2 Selected bond distances(Å) and bond angles (º) for POVs **1-2**

3. Figure S1 The FT-IR spectra data for POVs-**1**.

4. Figure S2 The FT-IR spectra data for POVs-**2**.

5. Figure S3 The PXRD patterns for POVs-**1** and POVs-**2**.

6. Figure S4 The PXRD patterns of POVs-**2** **(**Red**)** and calculated data (Black).

Table S1 Crystallographic data for POVs **1-2**.

	POVs-**1**	POVs-**2**
Formula	$C_{40}H_{50}Ni_2N_{16}O_{15}V_4$	$C_{40}H_{50}Mn_2N_{16}O_{15}V_4$
Fw	1316.14	1308.60
temperature/K	298.15	298.15
crystal system	monoclinic	monoclinic
space group	$P2_1/n$	$P2_1/n$
a/Å	14.4800 (12)	14.4878(12)
b/Å	12.5458 (11)	12.5478(11)
c/Å	15.4319 (13)	15.4244(13)
α/deg	90	90
β/deg	106.784 (2)	106.756(2)
γ/deg	90	90
V/Å^3	2684.0 (4)	2684.9(4)
Z	2	2
Dc/g·cm^{-3}	1.629	1.619
μ/mm^{-1}	1.433	1.202
F(000)	1340.0	1328.0
$R_1[I>2\sigma(I)]$	0.0664	0.0570
$wR_{2b}[I > 2\sigma(I)]$	0.1860	0.1492
final R_{1a}(all data)	0.1039	0.1057
wR_{2b}(all data)	0.2195	0.1801
GOF of F^2	1.059	1.028
CCDC. No	2279426	2279427

Table S2 Selected bond lengths [Å] and angles [º] for POVs **1-2**.

POVs-**1**			
V(1)-O(3)	1.616(4)	V(1)-O(2)	1.691(4)
V(1)-O(4)	1.768(4)	V(1)-O(5)	1.773(4)
V(1)-O(4)	1.768(4)	V(1)-O(3)	1.616(4)
V(2)-O(5)	1.806(4)	V2)-O(4)[1]	1.660(8)
V(2)-O(7)	1.617(5)	V(2)-O(6)	1.612(5)
Ni(1)-O(2)	2.050(4)	Ni(1)- N(3)	2.105(6)
Ni(1)- N(1)	2.116(6)	Ni(1)- N(5)	2.111(6)
O(2)-V(1)-O(5)	108.2(2)	O(2)-V(1)-O(4)	110.2(2)
O(4)-V(1)-O(5)	110.0(2)	O(3)-V(1)-O(5)	112.3(3)
O(3)-V(1)-O(4)	107.9(2)	O(5)-V(2)-O(4)[1]	112.1(2)
O(7)-V(2)-O(5)	110.2(3)	O(7)-V(2)-O(4)[1]	108.0(2)
O(6)-V(2)-O(5)	110.3(3)	O(6)-V(2)-O(4)[1]	108.3(3)
O(6)-V(2)-O(7)	108.0(3)	O(2)-Ni(1)-N(3)	86.74(19)
O(2)-Ni(1)-N(7)	84.91(18)	O(2)-Ni(1)-N(1)	85.21(19)
O(2)-Ni(1)-N(5)	88.3(2)	N(3)-Ni(1)-N(7)	92.3(2)
N(3)-Ni(1)-N(1)	171.5(2)	N(3)-Ni(1)-N(5)	86.7(2)
N(7)-Ni(1)-N(1)	89.7(2)	N(7)-Ni(1)-N(5)	173.2(2)
O(1)-Ni(1)-O(2)	177.6(2)	O(1)-Ni(1)-N(3)	94.5(3)
O(1)-Ni(1)-N(7)	93.0(2)	O(1)-Ni(1)-N(1)	93.6(3)
O(1)-Ni(1)-N(5)	93.8(3)	N(5)-Ni(1)-N(1)	90.3(2)

POVs-**2**			
V(1)-O(1)	1.689(3)	V(1)-O(4)	1.776(4)
V(1)-O(3)	1.771(4)	V(1)-O(2)	1.617(4)
V(2)-O(4)	1.807(4)	V(2)-O(3)^{1}	1.824(4)
V(2)-O(5)	1.614(4)	V2)-O(6)^{1}	1.613(5)
Mn(1)-O(1)	2.054(3)	Mn(1)-N(5)	2.112(5)
Mn(1)-N(7)	2.107(5)	Mn(1)-N(1)	2.113(5)
Mn(1)-O(7)	1.602(4)	Mn(1)- N(3)	2.108(5)
O(1)-V(1)-O(4)	108.21(18)	O(1)-V(1)-O(3)	110.14(17)
O(3)-V(1)-O(4)	109.93(18)	O(2)-V(1)-O(4)	112.1(2)
O(2)-V(1)-O(3)	108.1(2)	O(4)-V(2)-O(3)^{1}	112.07(19)
O(5)-V(2)-O(4)	110.3(2)	O(5)-V(2)-O(3)^{1}	107.9(2)
O(6)-V(2)-O(4)	110.4(2)	O(6)-V(2)-O(3)^{1}	108.0(3)
O(1)-Mn(1)-N(5)	85.13(16)	O(1)-Mn(1)-N(7)	86.87(16)
O(1)-Mn(1)-N(1)	85.61(16)	O(1)-Mn(1)-N(3)	88.54(17)
N(5)-Mn(1)-N(1)	89.88(19)	N(7)-Mn(1)-N(5)	92.4(18)
N(7)-Mn(1)-N(1)	171.93(18)	N(7)-Mn(1)-N(3)	86.56(19)
O(7)-Mn(1)-O(1)	177.78(18)	O(7)-Mn(1)-N(5)	92.9(2)
O(7)-Mn(1)-N(7)	94.2(2)	O(7)-Mn(1)-N(1)	93.4(2)
O(7)-Mn(1)-N(3)	93.5(2)	N(3)-Mn(1)-N(5)	173.62(18)
N(3)-Mn(1)-N(1)	90.31(19)		

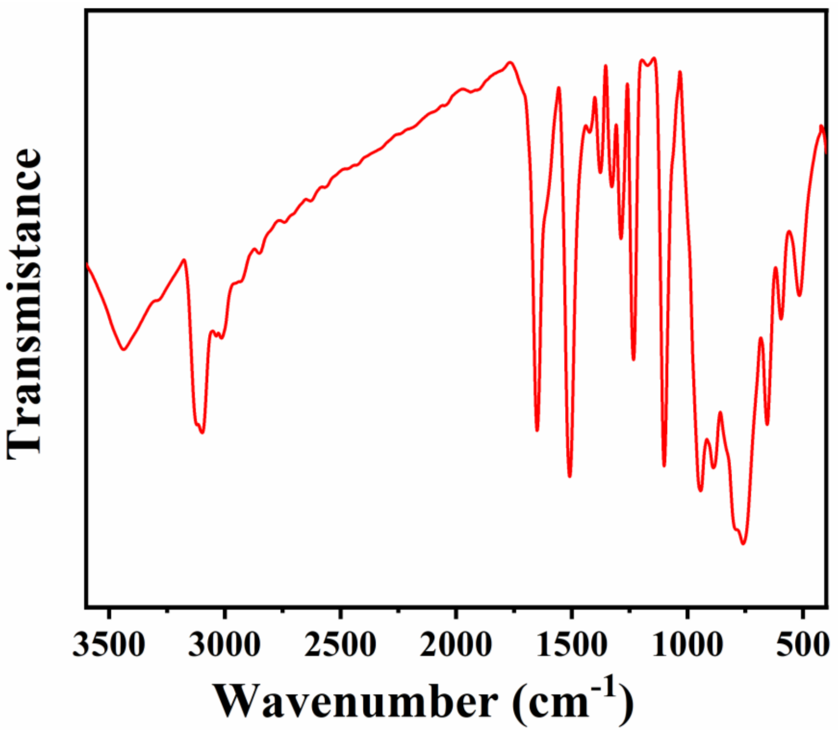

Figure. S1 The FT-IR spectrum of POVs-**1**.

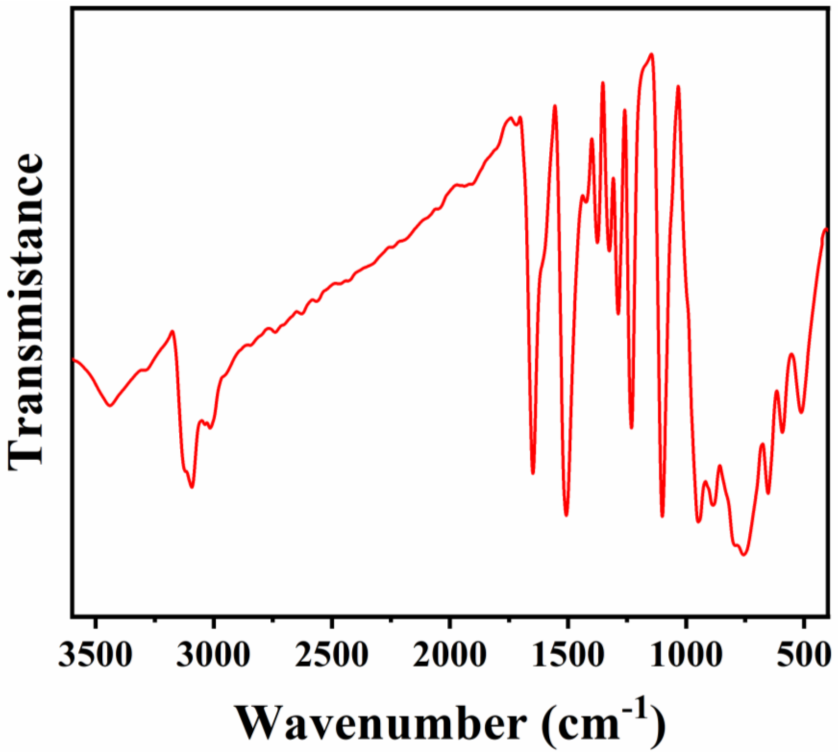

Figure. S2 The FT-IR spectrum of POVs-**2**.

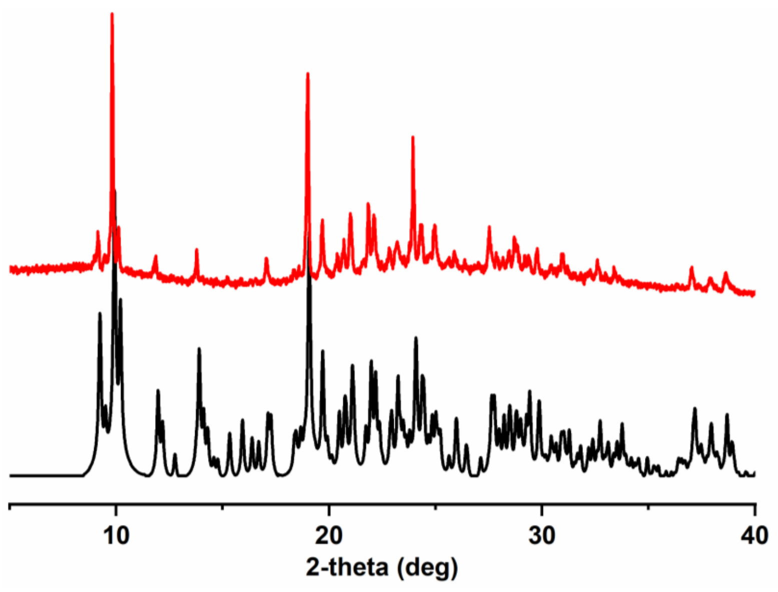

Figure. S3 The PXRD patterns of POVs-**1** (Red) and calculated data (Black).

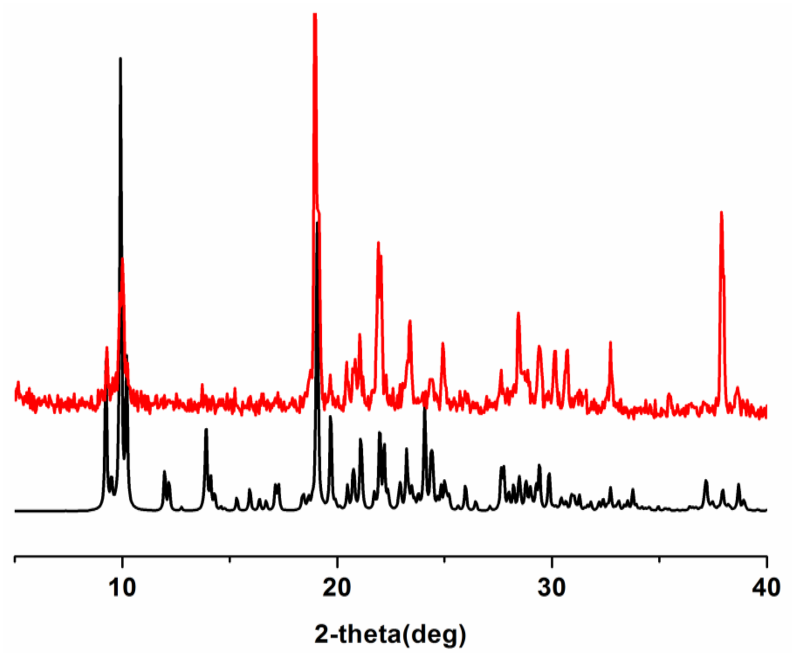

Figure. S4 The PXRD patterns of POVs-**2** (Red) and calculated data (Black).

Reference

[47] G. M. Sheldrick, *Acta Crystallogr. C: Struct. Chem.*, **2015**, 71, 3-8.
